# Hints for Invalids about to Visit Naples

**Published:** 1841-07-01

**Authors:** 


### HINTS FOR INVALIDS ABOUT TO VISIT NAPLES; BEING A SKETCH OF THE MEDICAL TOPOGRAPHY OF THE CITY, AND ALSO AN ACCOUNT OF THE MINERAL WATERS OF THE BAY OF NAPLES, &c. By *J. C. Cox*, M.D. 8vo. pp. 190, Longmans, 1841.

WE apprehend that it is with climates as with Spas—the air of the one, and the waters of the other, being over-estimated by those physicians who reside or practise on the spot. Dr. Cox assures us that this is not the case with him—that he is rigidly impartial, and without the slightest bias or prejudice in favour of the beautiful city and bay of Naples. We acquit him of all intentional embellishment or wilful exaggeration; but when we bear in mind that the Doctor owes his own life, and that of some of his family, to the climate described, we can hardly avoid the conclusion, that *gratitude* has disposed him to look with a favourable eye on the bright side of the picture, and draw a shade over the ruder features of the scene. Dr. Cox, however, is a sensible and intelligent investigator of his subject, and we are inclined to attach much confidence to his statements.

Our author has now resided some years in Southern Italy, and, as we before observed, has derived, together with his family, great benefit from the climate of Naples and vicinity. The superior longevity of the inhabitants of Great Britain, as compared with those of Italy, and, indeed, all parts of the Continent, presented a stumbling block on the very threshold of our author's inquiries. He has got over it in the following ingenious way.

"It may be alleged that the rate of mortality is less in Great Britain than in most other countries; and that *that* country must be the most healthy in which the fewest die. *But the physician has to do,* NOT *with the rate, but with the exceptions.*"

The meaning of this is, that if the inhabitants of Great Britain were transplanted to Italy, the rate of mortality would be increased; but that certain constitutions and maladies, forming the exceptions, are benefited by a change from the fogs and rains of England to the brilliant skies of Italy. To this position we see no objection.

The beautiful city of Naples, the ancient Parthenope, is divided into two quarters or climates by the Pizzo-Falcone hill, which extends into the sea, with an ancient castle on its most projecting point. The finest climate is possessed by the division, between Posilipo and the Falcone, stretching about the bay, and facing the south. Here the chief visitors reside, defended from the westerly and easterly winds by a range of hills, on the crest of which is placed the Castle of St. Elmo—the ornament and defence of Naples. This plain, formerly a marsh, is now well drained and fruitful. Naples has a mean temperature of 61° Fahr. and is refreshed by sea and land breezes during the summer months. Fine weather is said to be continuous from April till November, interrupted only by the solstitial and autumnal rains. The temperature is rarely above 86° or 88° at noon in the shade. In Winter, the temperature is seldom below 40° to 44° at night. The following table exhibits the mean average of the thermometer for the several months in the year:—

| MONTH. | Mean Temperature. | Number of Rainy Days. | MONTH. | Mean Temperature. | Number of Rainy Days. |
|---|---|---|---|---|---|
| January.... | 45° | 6 | July ...... | 76° | 3 |
| February ... | 48° | 7 | August .... | 76° | 4 |
| March ..... | 52° | 5 | September.. | 71° | 8 |
| April ...... | 57° | 8 | October.... | 62° | 7 |
| May ...... | 66° | 6 | November.. | 55° | 3 |
| June ...... | 70° | none | December .. | 51° | 8 |
| Average number of rainy days 65. Mean Temperature Fahrenheit 61. | | | | | |

The Appenines are not covered with snow till the middle of February, from which time till the melting of the snows, Naples is liable to the cold tramontane winds—a condition to which Naples is not alone exposed, since Nice, Genoa, Bologna, Florence, and even Rome itself, share the same scourges. The sirocco is still worse than the tramontane.

"The effect of the Sirocco, even at Naples, is very oppressive to the nervous system during the heat of summer, although it is not felt there nearly so severely as at Palermo. All nature seems to suffer from its influence, the air is still, the sea calm and leaden, the vine and the fig tree hang their leaves, animals are languid and oppressed, and the whole atmosphere is in a highly electric state. How changed are the sensations, when this electric condition is altered! the lightning flashes from mountain to mountain; Vesuvius, that mighty agent of electric influence, appears to be the centre of its action: the thunder rolls in terrific peals, increased in a tenfold degree by reverberation; then falls the rain, and all nature is refreshed; the depression of the nervous system ceases, and is succeeded by a delicious state of excitement and exhilaration.

These storms have a great and painful effect upon delicate and susceptible nervous subjects, but the change of weather removes the irritation or depression, and restores the *bien etre*." 16.

While writing these lines we were visited by a nobleman, who, with his lady, had resided in Naples during the last Winter, 1840-1. They both declared, that no earthly consideration would induce them to encounter again the siroccos and tramontanes of Naples. The gentleman had gone to Italy for his health and returned worse than when he left home.

"The soil of Naples is of a light sandy character, and resting upon a porous Tufa, through which water readily percolates. Hence the effects of rain quickly pass away, and as soon as the storm subsides, the invalid may with safety walk in the Villa Reale. Wells are easily made in this tufous rock, and water is found very near the surface; but it is brackish and unwholesome, producing diarrhœa and dysentery in strangers." 19.

Care should be taken by foreigners that water is procured from the fountains, and not from the wells of Naples. The markets are well supplied with wholesome food at a moderate price.

"The effect of the neighbourhood of a volcano on the human frame is very little observed or understood. That a very powerful electric action is going on in

Vesuvius, and other volcanos, no one can doubt. The constant change that takes place in the chemical relation of bodies acted on by it, must necessarily cause the evolution of electricity to a prodigious extent. These phenomena become sometimes cognizable to our senses; clouds are formed over the cone, or attracted to its side, from which they are awhile suspended, and then are detached and repelled. Sometimes dark volumes of cloud collect over the horizon, while the thunder pealing in the distance indicates the rapid approach of storm. Attracted by the mountain, the lightning flashes down the crater, while the fearful rolling of the clouds encircling it reverberates in oft repeated echoes from its sides. A calm succeeds, and all is dispersed. What is this but the electric agency of the mountain? The effect on some susceptible and nervous subjects is very striking, causing headache, general nervous pains, great irritability of the system, sleeplessness, and an almost irrepressible degree of excitement. The influence of so powerful an electric agent in such cases, is manifestly injurious." 24.

The highly stimulatory air of Naples renders it a precarious, not to say dangerous, residence for people of irritable temperaments. The city itself is not subject to malaria, but many parts of the neighbourhood, as Averno, Agnano, and Fusaro, are very insalubrious, as is the whole plain west of Posilippo, and the country around Pozzuoli, Baiæ, and Cuma. The population of Naples is 380,000, the deaths one in thirty.

Dr. Cox, in his third chapter, takes up the question—"*Is Naples a good Winter residence?*" The months of September, October, November, December, and January, are stated by our author to be "beautiful, and of a temperature very favourable for an invalid." During these months, exercise may be taken in the exploration of the classic environs of this ancient capital. The months of February and March may, he thinks, be passed without danger, if common precautions are taken. During these months, the invalid should restrict his rides to the Riviera di Chiaja, the shores of the Bay of Baiæ, or the Strada Nuovo; return home by four o'clock, and shun evening assemblies. Great attention to dress is necessary.

"With moderate precaution then, the winter, if it may be so called, may be passed without inconvenience or danger; and during this season, Naples offers advantages as great, or greater than any city on the Continent. Rome has no walks so well defended as the Villa Reale; one side of the street often glowing with the heat of summer, while the other is cold and damp as winter. Florence is the least eligible of all places for the invalid in winter, though lovely and delightful in spring and autumn. Nice has the Maestrale, which is almost irrespirable. Pisa, perhaps, along the Lung Arno, is the most defended of any place; but, if the patient extend his walk beyond this limit, he is stricken with the cold blast; and in the *agrémens* of society both of Rome and Naples, it is very deficient." 31.

The Museo Bourbonico offers a good winter lounge; but it is to "be visited only with thick shoes, woollen socks, and a warm cloak." "Naples is not a desirable residence during July or August, excepting on the hill of the Vomero, or at Capo di Monte, above the city." The heat of the atmosphere reflected from the lava in the streets is excessive, and much more difficult to bear than that of the East and West Indies. Castellamare is then the refuge from Naples.

To the question—"What are the diseases benefited by the climate of Naples?"—Dr. Cox very properly answers—"Certainly not consumption in its latter stage."

"But there are many forms of bronchial phthisis in which the air of Italy effects great relief, and even removes the disease. Where severe bronchitis has existed, succeeded by continued cough, with suspicious expectoration, tendency to hectic fever, but with the chest tolerably free on percussion; mucous rattle without pectoriloquy, and no emaciation, in such cases the air of Italy often effects wonders.

In the winter's cough of old persons, with great bronchial irritation, the effect of the air of Naples is also most beneficial.

In cases of hæmoptœ, where has pre-existed irritable mucous membrane, only with slight hæmorrhage from the lungs or throat, and emaciation, such cases, indeed, as have been treated for consumption with severe and depressing remedies, the air of Naples is most specific. In cases of hæmoptœ with tubercles, or in hæmoptœ of an active kind, the air is too stimulating to effect more than a transient benefit.

Where there has been reason to fear incipient tubercular disease, manifested by slight hacking cough, with dullness on percussion over some part of the chest, tested by auscultation, and the general health delicate and languid, in such cases the pure marine air of Naples often dissipates the bad symptoms, and restores the health.

In those persons who suffer from bronchial irritation, associated with disordered health, from residence in hot climates, with biliary obstruction, and yellowness of the skin, Naples is a most desirable winter's residence; where the mineral waters of Castellamare are at least equal to those of Cheltenham, or even superior to them.

In some cases of humoral asthma it is very useful; while in other form of asthma the atmosphere is objectionable, and the air of Rome is more salutary.

In dyspeptic cases, of various grades and forms, Naples is usually beneficial; as also in hysteria, and hypochondriasis, with nervous debility, and depression of spirits; but when the latter symptom occurs in full and plethoric habits, its climate is too stimulating.

In many cases of neuralgia, especially those of rheumatic origin, the uniform temperature and tonic atmosphere of Naples in summer, are exceedingly useful. A lady who suffered severely from tic douloureux, was quite free from it, while she remained there.

In children suffering from scrofula, in whom the climate of England is constantly predisposing them to tracheal, or pulmonary inflammation, having enlarged tonsils, and irritable lungs, with a tendency to obstruction of the mesenteric glands, and general strumous predisposition, the dry marine and electro-tonic atmosphere of Naples acts as a charm." 40.

Our author thinks that the air of Naples is too stimulant in many cases of gout—that of Rome is preferable. The baths of Ischia are beneficial in many cases of rheumatism.

"The constant influence of a climate so stimulating as that of Naples, tends greatly to increase the general irritability of the system. To those who come only as occasional visitors this is less obvious; but, after living two or three years there, it becomes very evident. Our countrymen pursue their usual mode of living, instead of adopting the habits of the country in which they reside: they take a large proportion of animal food, and drink the strongest wines, in their accustomed quantity; hence, after being a few months exposed to the climate on this uncongenial diet, they are frequently attacked with fever, and sometimes with phrenitis. It is perhaps preferable to adopt, in some degree, the customs of the inhabitants of the countries in which we reside; and, in this instance, to take a larger quantity of vegetable, and less of animal food, and to reduce the quantity of stimulant." 43.

The foregoing observations of our author, though probably a little tinged in favour of his darling Italy, are, upon the whole, judicious, and are worthy of attention on this side of the channel.

### MINERAL WATERS.

Naples and its vicinity present some important spas. Those in Naples are the Acqua Solfurea di Santa Lucia, and Acqua Ferrata di Pizzo-Falcone. The former is a simple sulphur water, analogous to that of Moffat, of limpid sparkling appearance, containing sulphuretted hydrogen gas. It is frequented in the months of June, July, and August. The Acqua Ferrata rises at the bottom of a cavern, brisk, and sparkling, decidedly chalybeate in taste, and a good deal resembling the water of Tunbridge, but containing much more carbonic acid. The medium dose is three pints, taken in the morning fasting, with intervals of exercise. Its reputation is very extensive in Italy.

The Acqua Bagnuoli rises on the shore of the bay of Baiæ, two miles from Naples, beyond the Grotto of Posillippo. It contains 38 grains of saline matters in the pint—chiefly carbonate and muriate of soda, and has a temperature of 107°. It is chiefly used in baths; but is also taken internally.

A most powerfully astringent water springs up in the crater of Solfaterra. It is of a harsh styptic taste, and exceedingly astringent. It is used by the poor of the neighbourhood in leucorrhœa, blennorrhœa, hemorrhoids, passive hæmorrhages, dysentery, &c. The pint contains 21 grains of alum, and five of sulphate of iron, besides many other ingredients!

Near Pozzuoli there is a spring nearly resembling the waters of Weisbaden, "*which are red and stain the skin*" a fact that we have never before observed, though we have often dipped into the waters of the Kockbrunnen.

Our author gives an account of the mineral waters of Pozzuoli, where one of them is situated in the ancient temple of Serapis. The thermal springs here shew a temperature of 106°—in colour transparent—taste slightly saline—odourless. They contain 29 grains of saline matters in the pint—11 of which are carbonate of soda—2 carb. magnesia—4½ sulphate of soda—9 muriate of soda—half a grain of carbonate of iron. They are considered analogous to the waters of Teplitz; but this is a great mistake, for the Teplitz waters contain only five grains in the pint, two or three of which are carbonate of soda. The cold springs of Serapis contain about 45 grains in the pint, 24 of which are muriate of soda—ten of carbonate of soda. They are represented as analogous to the waters of Ems.

Still farther on the road to Baiæ, beyond the Lucrine lake, are the baths of Nero, issuing from a cavern on the side of a hill, something like Pfeffers in the country of the Grisons. The cavern is filled with steam and the source is at a temperature of 180°—considerably higher than the Sprudel, at Carlsbad. The cavern itself is almost too hot to bear, producing quick and copious perspiration.

These are the principal mineral waters which exist in Naples and its neighbourhood. In this short circuit are found waters of different composition, sulphurous, chalybeate, aluminous, saline, alkaline, and acidulous; of temperatures varying from 60° to 180° of Fahrenheit. These varied springs are found in a country of the most picturesque character, of a climate exceedingly delicious, and in situations full of classic and scientific interest." 62.

### Ischia.

This is one of the most interesting of the Mediterranean Islands, whether we regard the beauty of the scenery, the softness of the climate, the fertility of the soil, or its geological structure. Its thermal waters are of great variety in temperature and composition, abounding in saline materials and gaseous impregnations. The island is 20 miles South West of Naples, ten miles from Misenum, and presents the appearance of a conical mountain rising out of the sea 2450 feet. It is like a garden covered with vines, and tempered by the sea. It bears the marks of an extinct volcano, the last eruption of which occurred more than 500 years ago. Hot springs exist here, of various temperatures, up to more than 200° of Fahrenheit. Hot vapour also rises in various places from the earth. The sands on different parts of the coast are almost too hot for the hand—even in the sea, where bathing is practised. The island presents great facilities for sea-bathing. The most important spring on the island is *Gurgitello,* in the valley of Ombrasco, and furnishes water in abundance.

"There is a large bath-room, containing seventy-six baths, of which ten are fitted up for applying warm douches to any part of the body; and also a capacious sudatorium, or vapor bath, which is not, however, completed. There are twenty-one private baths, moderately well kept." 83.

The water is clear to the sight, unctuous to the touch, and rather nauseous and saline to the taste. It contains abundance of carbonic acid gas. Mud-baths are established here, at a temperature of 128°, consisting of sediment, carbonate of lime, and other earthy matters. Each pint of this spring, which shews a temperature of 158°, contains 40 grains of saline matters, of which 20 are common salt, 18 carbonate of soda, the other two being made up of various salts, with a mere trace of iron. It is considered analogous to Carlsbad, though the latter contains 20 grains of sulphate of soda, and the former none. The Carlsbad waters shew ten grains of carbonate of lime, whereas those of Gurgitello contain little more than half a grain.

"The Acqua Gurgitello is administered both internally and externally. Invalids usually take two or three pints in the morning, with intervals of half an hour between each glass, which are best employed in exercise. The baths must be used under the direction of one of the physicians of the island, who are best acquainted with the peculiar effects of the course. As is common during the employment of mineral waters of this powerful character, a certain degree of constitutional change is produced in a shorter or longer period, which must be watched and carefully regulated; although it is rarely so great as to require more than a partial suspension of their use. This effect ordinarily precedes, and is commonly attended by, some alleviation of the malady; hence it is probably produced by the absorption of the mineral water into the system. It is better to take two courses of the baths, of four or five weeks each, leaving an interval of ten or fifteen days for the invalid to recruit his strength." 87.

This "BAD-STURM," we apprehend, has been too often the effect of inattention to the bowels of the spa-bibbers, rather than to a salutary operation of the waters. If it be a *salutary* crisis, it surely ought not to be checked—if not, it ought to be prevented. We leave the Spa Doctors to choose which horn of the dilemma they may prefer.

"The waters of Gurgitello have received ample testimony of their efficacy in various diseases; in gunshot wounds; in fistula; in rheumatic affections; in the sequelæ of gout; in old ulcers; in uterine diseases, as dysmenorrhea, amenorrhea, irritable uterus, and sterility consequent on that state; in paralysis, especially as the result of fever; in glandular and other scrofulous diseases; in dropsical effusions, arising from exposure to cold or humidity; in chronic peritonitis, and other chronic inflammations; in contractions of the limbs; visceral obstructions, as of the liver, from living in hot climates; and of the spleen, or pancreas, the result of intermittent fever; in obstruction of the mesenteric glands; in irritable bladder; and nephritic disorders; in repelled eruptions; in pains of the bones, and other syphilitic affections, or the consequences of mercurialization; in old Indians who have been injured by climate and excesses, in whom the effects of the climate of England are often prejudicial; in the man of dissipation, whose constitution has been debilitated and impaired by indulgence; in all these cases, the baths of Gurgitello, and the beautiful atmosphere, and tranquil seclusion of Ischia, are most strikingly beneficial. But there are neither gaming tables nor balls; the short period of sojourn there is devoted solely to the recovery of health; and those who have other objects, may drink the water of Baden-Baden, or Wiesbaden." 90.

The Acqua dell' Occhio "is smooth and agreeable to the skin, and has an exceedingly pleasant effect upon the surface, similar to the waters of Langen-Schwalbach." Here the worthy doctor has made a great mistake. Langen-Schwalbach is a strong chalybeate, corrugating instead of smoothing the skin. The waters which bear any analogy to those of OCCHIO, are *Schlangenbad.*

We must pass over at least a dozen of other mineral waters springing out of the soil, or rather the lava of Ischia.

"In various parts of the island of Ischia are fumaroles, which are apertures in the soil, from which hot air or vapor is emitted. In certain places these are made subservient to medical purposes; small buildings are erected over the fumarole, and earthen pipes so arranged as to conduct the vapor to different parts of the body. These stufe, as they are are termed, constitute most valuable natural vapor, or hot air baths, which are applicable to various disorders. The vapor is not, however, in any degree medicated, but is simply the vapor of water, as the condensed fluid clearly shews. Nor is the hot air medicated with any particular gas, or volatile body, but is simply atmospheric air heated to a given point. The vapor is emitted at a temperature of from 130° to 140° of Fahrenheit; but the quantity may be modified so as to adapt the temperature of the room to the malady and the feelings of the patient." 130.

"How wonderful then, and how numerous are the medical resources of this extraordinary country. Surely no spot of fifteen miles in circumference ever afforded so vast a series of interesting phenomena. The Ischiote girl boils her vegetable meal in a stream, hot at its source; and, in the heating of it, a fire is employed, compared with which, that which burns for the preparation of the grandest city entertainment is but as a spark. The vine dresser, returning in the evening from his toil in the southern sun, cuts a trench with his hoe in the sand on the sea shore; in a moment it is filled with a hot and mineralised water, in which he bathes his feet, swoln and tender from the rugged stones and scorching heat to which they have been all day exposed, bare and unprotected. He sits on the ground; and, while enjoying his luxurious pediluvium, he carols his airs, with all the liveliness and gaiety so characteristic of the Ischiote peasantry; and after a quarter of an hour's bath, he pursues his way home, refreshed and recruited. The fire which warms his foot-bath is the same as that which kindles Vesuvius, and which bursts not in Ischia, only because it has its exit elsewhere. The matron needs no other fuel to heat the water in which she washes the linen of her family, Nature has supplied her with an inexhaustible abundance, at a temperature hot as her hands can bear, and ready charged with alkaline ley from the bowels of the earth." 136.

### Castellamare.

At the eastern corner of the bay of Naples, and at the base of an Appennine-ridge, lies Castellamare—the Ramsgate of Naples, containing 24,000 inhabitants. It was overwhelmed with lava and ashes from Vesuvius in 79, when Pompeii and Herculaneum were buried. Though the town is built on the level of the sea, yet numerous villas are scattered up the neighbouring acclivities for the accommodation of visitors. The temperature of the air here is eight degrees lower than at Naples in the day, and ten or twelve at night. There is no malaria around Castellamare.

The mineral waters of Castellamare are numerous and various. They rise from the limestone rock at the foot of Monte Gauro, and have a temperature of 65°.

Some of them are sulphureous, and most of them chalybeates—almost all combined with salines—the proportions of which Dr. Cox gives in separate tables. Thus, the acqua solfurea del' muraglione contains 70½ grains of solid matters, of which 42 grains are muriate of soda—6 bi-carbonate of soda—2 carb. magnesia—2 carb. lime—4 sulphate of soda—5 muriate of lime—3 muriat. magnesia, with some minor materials, including traces of iron.

"The properties of this mineral water are very important. In obstructions of the viscera, of the liver, spleen, pancreas, or mesenteric glands, in obstinate constipation, in hæmorrhoidal affections, in all diseases of plethora, in apoplectic diathesis, in epileptic tendencies, in obesity, in all those cases this water is often exceedingly valuable; and also in cutaneous diseases, in suppressed eruptions, in chronic psora, used both externally and internally, it is very beneficial. Its action on the bowels, in clearing out the intestinal canal, is prompt and efficacious. When taken in moderate doses for some time, it unloads the portal system and produces a feeling of lightness and of health. As, however, its action is often considerable, producing very profuse evacuations, it should be used with caution, and not in great quantities, excepting under proper direction. It is beneficial in gouty cases of a tonic character, but it is not proper in atonic gout, when the patient is much debilitated. The power of this water in some cases of dropsical accumulation is very striking; the discharge of large quantities of liquid evacuations, and great diuretic effect, fulfil two very obvious indications in these disorders, and have the happiest results, especially being combined with a pure and tonic atmosphere. It is peculiarly celebrated for relieving obesity, to which the Italians are much disposed from their indolent and sedentary habits, doubtless induced by climate, and which is especially observable in females. There are few mineral waters so rich in saline constituents as the two Acqua del Muraglione, and scarcely any in which the medical effects are more decidedly useful, when properly administered." 173.

Dr. Cox concludes thus :—

"We have now given a rapid sketch of the mineral waters of Castellamare, and have shewn that they are analogous and equal to the most celebrated waters of Spa, Pyrmont, Seltzer, Tunbridge, Leamington, Kissingen, Cheltenham, Harrogate, Schwalbach, Ems, and Tœplitz; that they are in many instances superior, as being richer in saline ingredients, and that they are in abundant quantity and occurring in a most lovely climate. It has also been shewn that at Ischia, and at Pozzuoli, thermal springs in great profusion and of the highest character abound, in strict analogy, both in temperature and composition, to the distinguished baths in Germany, Carlsbad, Wiesbaden, Baden-baden, Wildbad, and Gastein; and that there are others which are of very rare and important chemical composition." 176.

If the above representation be correct, and we have no reason to doubt its accuracy, a new field is open to our spa-goers, many of whom would prefer the beautiful shores of Italy in the Summer to a long journey over the Alps from Italy, to the spas of Germany. It is on this account that we have given a fuller review of this volume than we otherwise would have done, in order that medical men in this country may be able to communicate important information to such of their patients as are proceeding to Italy for their health. The volume itself may be safely recommended to the invalid, as a topographical as well as medical guide to Naples and its vicinity.

